# One Advantage of Marriage

**Published:** 1843-12

**Authors:** 


*One Advantage of Marriage.*—Dr. Casper, of Berlin, has estimated the mortality of unmarried male individuals, between the ages of 30 and 45 years, to be 27 per cent., while among married men of the same period of life it is only 18 per cent. For 41 unmarried men, 78 married men attain the age of 40 years; and the disparity is more striking as age advances; for at 60 years of age there are only 22 bachelors to 48 Benedicts; at 70, 11 to 27; and at 80, 3 to 9.—*Ibid,* from *Gazette des Hôpitaux.*

